# Functional Bowel Disorders: A Retrospective Descriptive Study in Dakar, Senegal

**DOI:** 10.7759/cureus.103082

**Published:** 2026-02-06

**Authors:** Abdel Aziz Atteib Fall, Mamadou Ngoné Gueye, Mama Ndieme Diouf, Bibata Toure, Salamata Diallo, Daouda Dia

**Affiliations:** 1 Gastroenterology and Hepatology, Cheikh Ahmadoul Khadim National Hospital, Touba, SEN; 2 Gastroenterology and Hepatology, Idrissa Pouye General Hospital, Cheikh Anta Diop University, Dakar, SEN; 3 Gastroenterology and Hepatology, Aristide Le Dantec Hospital, Cheikh Anta Diop University, Dakar, SEN

**Keywords:** adult gastroenterology, functional bowel disorders, irritable bowel syndrome, rome iv criteria, senegal

## Abstract

Introduction

Functional bowel disorders (FBDs) are common pathologies worldwide. However, little is known about the subject in our region. Thus, we undertook a cross-sectional study to address the issue in our population.

Methodology

We conducted a retrospective descriptive study from January 2016 to December 2022 using data from 2,831 outpatients seen in the hepatogastroenterology department of Idrissa Pouye General Hospital in Dakar. All cases consistent with an FBD according to the Rome IV criteria were included. We collected and analyzed epidemiological, diagnostic, therapeutic, and evolutionary data.

Results

We included 397 of 2,831 patients (14.02%), with a mean age of 43.23 years and a sex ratio of 0.91. Symptoms were dominated by abdominal pain, which was present in 273 cases (68.8%). The constipation-predominant subtype of irritable bowel syndrome was the most common, found in 273 cases (32.2%). Dyspepsia was the most frequent associated symptom, reported in 102 cases (25.7%). The mean duration of disease at diagnosis was 5.72 years. Anxiety was associated in 11 cases (2.77%), and physical examination findings were normal in 296 cases (74.6%). Additional investigations were performed in 230 patients (57.9%). Treatment was mainly based on antispasmodics, prescribed in 236 cases (59.45%), and defoaming agents, prescribed in 303 cases (76.32%). Clinical evolution was marked by an improvement in symptoms in 183 cases (46%).

Conclusions

In our cohort, FBD mainly affected young adults, with a female predominance. Abdominal pain was the most common manifestation. Treatment was symptomatic, based on defoaming agents and antispasmodics, and a favorable outcome was observed in nearly half of the patients.

## Introduction

Functional bowel disorders (FBDs) are syndromes associated with abdominal pain, bloating, and/or changes in bowel habits. They are integrated into disorders of gut-brain interaction, as defined by Rome IV, formerly known as functional digestive disorders (FDDs).

Indeed, under the name FDDs, a set of various syndromes is found that brings together isolated or associated digestive symptoms, which evolve chronically, continuously, or intermittently, without an organic cause detectable by routine examinations [1,2].

Dietary risk factors (incompletely absorbed sugars, fatty foods, spices, alcohol, coffee, etc.), psychological (childhood trauma), lifestyle (smoking, lack of physical activity), and genetic factors are implicated in the occurrence of these disorders [3-8]. They are believed to be the cause of alterations in digestive sensitivity and motor skills, neuropsychic influences, and abnormalities of the intestinal microbiota, which explain the symptoms. However, its pathophysiology remains incompletely elucidated [9,10].

These pathologies affect the entire digestive tract and include FBDs. They are classified according to the Rome criteria.

The global prevalence of FDDs is approximately 43%, and that of FBDs is 35.6% [11].

In Africa, and particularly in Senegal, data on this condition are limited. Based on this observation, we conducted this study to identify the sociodemographic, diagnostic, and therapeutic characteristics of FBDs in our population.

## Materials and methods

Study design

We conducted a retrospective descriptive study between January 2016 and December 2022, a period of seven years.

The study was conducted in the outpatient unit of the Internal Medicine and Hepatogastroenterology Department at Idrissa Pouye General Hospital in Dakar, a level-3 hospital according to Senegal’s health reference system.

Study population

Our population consisted of all patients seen in consultation at the outpatient unit during the study period.

Inclusion criteria

We collected the records of patients whose diagnosis was compatible with a functional bowel disorder according to the Rome IV criteria, namely main symptoms such as abdominal pain, changes in bowel habits, or abdominal distension/bloating, evolving over at least six months and occurring at least one day per week during the past three months.

Exclusion criteria

We did not include records of patients in whom an organic lesion was discovered during follow-up, those with functional diarrhea who had not undergone a total colonoscopy with biopsies and pathological examination, patients with incomplete information on age or sex, or records that were lost at any point during follow-up.

Data collection and analysis techniques and tools

We collected data on age, sex, symptoms, results of biological, radiological, and endoscopic assessments, therapeutic options, and the patients’ evolving clinical profile.

Data entry and analysis were performed using Excel (version 15.41) and SPSS (version 18, SPSS Inc., Chicago, IL) software.

## Results

**Figure 1:**
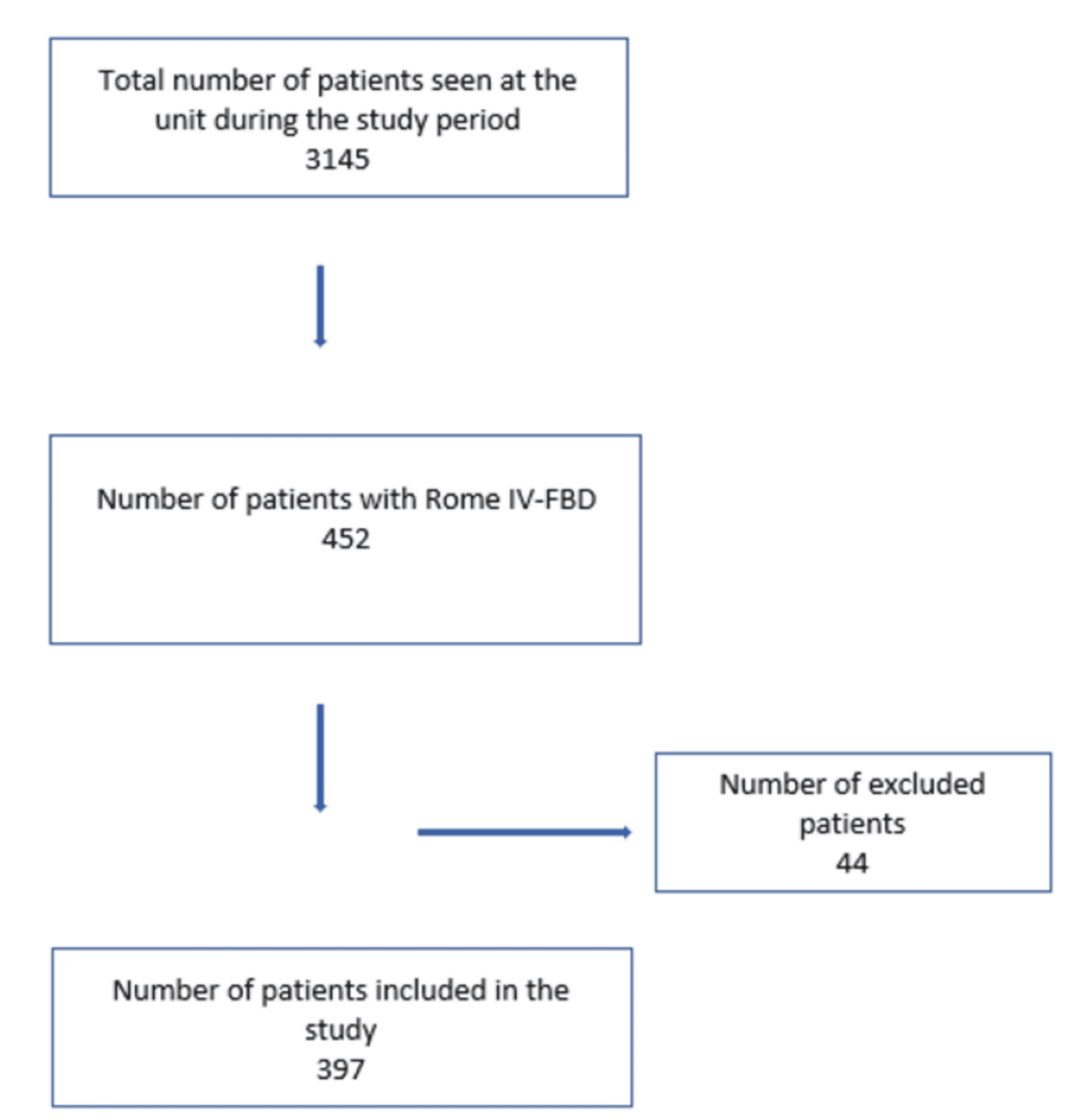
Flow diagram. FBD, functional bowel disorder Image credit: Atteib Fall.

We included 397 records out of 2,831, representing a hospital-based prevalence of 14.02% (Figure 1). There was a female predominance, with a sex ratio of 0.91 (208 women). The mean age of our patients was 43.23 years, with the 30-45 age group accounting for 144 cases (36.3%).

The medical history of our patients was mainly metabolic pathologies, such as hypertension in 40 cases (9.6%), metabolic syndrome in 25 cases (4.5%), and diabetes in 8 cases (1.5%) (Table 1).

**Table 1:** Medical history of the patients (N = 397).

Antecedent	Number of patients (%)
Hypertension	40 (9.6)
Metabolic syndrome	25 (6.3)
Hepatitis B virus infection	12 (2.5)
Abdominal surgery	9 (2.2)
Asthma	8 (2)
History of abortion	8 (1.8)
Diabetes	8 (1.8)
Sickle cell trait	7 (1.5)
Helicobacter pylori gastritis	3 (0.8)
Hemorrhoidal disease	3 (0.8)
Fatty liver	3 (0.8)

A history of smoking was present in 14 cases (3.5%), alcoholism in 6 cases (1.5%), and coffee consumption in 15 cases (3.8%).

**Figure 2:**
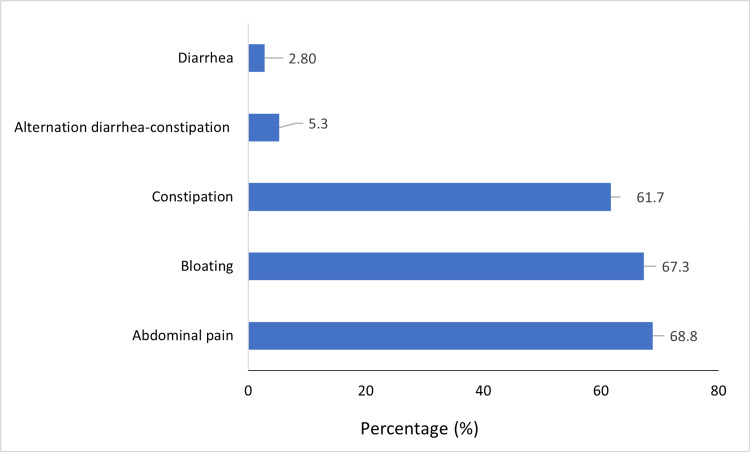
Frequencies of different FBD symptoms (N = 397). FBD, functional bowel disorder

The symptomatology of FBD was dominated by abdominal pain in 273 cases (68.8%) and bloating in 267 cases (67.3%) (Figure 2).

Dyspepsia was the most common associated digestive sign found in 102 cases (25.7%), followed by gastroesophageal reflux disease (GERD) in 91 cases (22.9%), heartburn in 56 cases (14.11%), and the occurrence of new-onset hemorrhoids in 45 cases (11.34%). The associated extra-digestive signs were pain syndromes (headache, diffuse pain, arthralgia) in 42 cases (10.57%), anxiety in 11 cases (2.77%), and insomnia in 9 cases (2.27%). The physical examination was normal in 296 cases (74.6%) and found abdominal meteorism in 58 cases (14.6%).

Additional explorations were carried out in 230 cases (57.9%). Those were biological examinations in 42 cases (10.6%) and morphological (abdominal ultrasound, colonoscopy, upper endoscopy, abdominal CT) in 227 cases (57.2%). One case of hyperglycemia and one case of Entamoeba histolytica cysts were identified. Colonoscopy, prescribed in 47 cases (11.59%), was normal in 44 cases, in favor of uncomplicated colonic diverticulosis in one case, and found polyps in two cases. Abdominal imaging prescribed in 141 cases (35.52%) was normal in 59 cases, found aerocolia in 56 cases, and abnormalities unrelated to the clinical picture in the rest of the patients. Upper endoscopy, performed in 115 cases (28.97%), revealed gastritis in 55 cases, with evidence of Helicobacter pylori in 31 cases; an incompetent cardia, hiatal hernia, and peptic esophagitis were found in 19, 12, and 2 cases, respectively.

Irritable bowel syndrome (IBS) was the most frequently found type of FBD, present in 160 cases (40.3%), with IBS-C found in 128 patients (32.2%). Functional constipation was the second most common, with 105 cases (26.4%), followed by functional bloating in 74 cases (18.6%) (Table 2).

**Table 2:** Different types of FBD. IBS, irritable bowel syndrome; IBS-C, constipation predominant; IBS-D, diarrhea predominant; IBS-I, undetermined subtype; IBS-M, mixed subtype; FC, functional constipation; FB, functional bloating; NSID, non-specified bowel disease

Type of FBD	Subtype	Number of patients (*N *= 397)	Overall prevalence (%)
IBS		160 (40.3)	5.16
	IBS-C	128 (32.2)	4.13
	IBS-D	14 (3.5)	0.45
	IBS-I	13 (3.3)	0.42
	IBS-M	5 (1.3)	0.16
FC		105 (26.4)	3.34
FB		74 (18.6)	2.39
NSID		58 (16.6)	1.87

**Figure 3:**
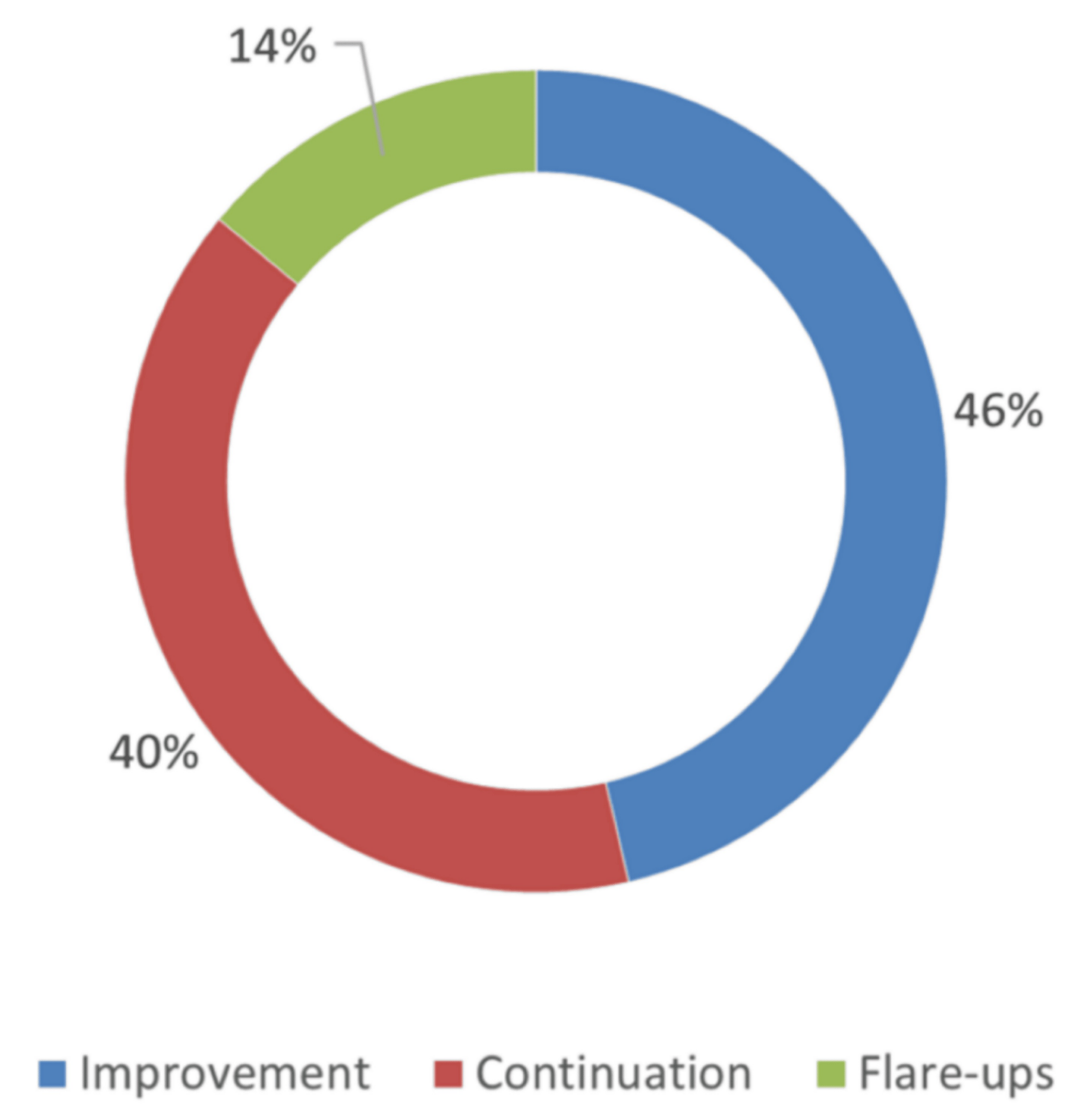
Evolution of the disease during follow-up (N = 206). Image credit: Atteib Fall.

The treatment administered included a defoamer in 303 cases (76.3%), an antispasmodic in 236 cases (59.5%), a laxative in 180 cases (45.7%), neuromodulators in 12 cases (3.02%), and rifaximine in 11 cases (2.8%). Dietary guidelines were associated with 94 cases (23.7%), and additional treatment based on proton pump inhibitor in 77 cases (28.1%), antacid in 47 cases (17.2%), and prokinetic drugs in 37 cases (13.5%). The median duration of symptoms was three years, ranging from 1 to 40 years; the median number of consultations in the department was 3, with a range of 1 to 27 consultations (Table 3). Among the 206 patients who were followed up, the outcome was favorable in 183 cases (46%) (Figure 3).

**Table 3:** Duration of the disease.

Duration (years)	Number of patients (%) (*N* = 397)
[1; 5[	150 (58.3)
[5; 10[	42 (16.3)
≥10	65 (25.4)
Total	257 (100)

## Discussion

Literature data on the prevalence of FBD are very heterogeneous across the globe. This is due to the variable nature of the study populations, the data collection methods, but above all, the diagnostic criteria.

The prevalence of FBD in our study was 14.02%. Among studies using the Rome IV criteria, similar results were reported by Norwood et al. in 2020 in Honduras (14.4%) [6]. However, Sperber et al. in 2020 reported a higher overall prevalence of 35.6% in the general population, figures similar to those reported by Aziz et al. in North America in 2017 (28.1%) [11,12].

In Africa, we did not find any studies on FBD based on the Rome IV criteria.

The lower prevalence in our study and that reported by Norwood et al., compared to that of Aziz et al., could be explained by differences in etiopathogenic factors (dietary, psychosocial, microbiota-related, genetic, etc.) between countries of the Global South and the Global North. Under-recognition cannot be ruled out [6,12].

IBS is the most studied type of FBD and was the most common type in our study, with a prevalence of 5.2%. Sperber et al. reported an overall prevalence of 4.1%, which is comparable [11].

The mean age of the patients was 43.23 years (range: 11-87 years). FDDs are primarily diseases of young adults, and their frequency decreases with age [2,12].

We noted in some cases dysmetabolic pathologies (17.4%), including hypertension (9.6%), metabolic syndrome (4.5%), diabetes (1.5%), overweight (1%), and dyslipidemia (0.8%). This is a fairly new concept reported by a few studies. Guo et al. in China found an association of IBS with metabolic syndrome and hypertriglyceridemia [3]. Gulcan et al. in Türkiye observed an association with prediabetes and dyslipidemia [13]. This association can be explained by the effects of disturbances in the intestinal microbiota, which constitutes a pathophysiological element found both in FDDs and in metabolic syndrome, and which could thus constitute a bridge between the two diseases [3,10,14].

A history of abdominal surgery was present in 2.2% of patients. In fact, a higher frequency of abdominal surgery was found in individuals with FBD compared to the general population. We can take as an example the study by Longstreth and Yao, who reported a higher frequency of cholecystectomies, appendectomies, hysterectomies, and spinal surgeries in this population [14]. The studies by Kennedy and Jones, Ryle, and Chaudhary et al. corroborate these results [4,15,16].

The duration of symptoms was specified in 257 patients, with a median of three years, a mean of 5.72 years, and a range of 1 to 40 years. Variable average durations of symptom evolution are reported in the literature. For example, the Kenyan study by Lule and Amayo found a mean duration comparable to ours - 5.5 years (range: 1-36 years) - in patients with IBS [17]. These results highlight the chronic nature of functional intestinal disorders and their long-term progression.

Among the manifestations of FBD in our patients, abdominal pain was the most frequent, present in 68.8% of cases, followed by abdominal bloating (67.3%), constipation (61.7%), diarrhea-constipation alternation (5.3%), and diarrhea, which was the least frequent symptom (2.8%). These data are comparable to those reported in other studies, some of which are presented in Table 4.

**Table 4:** Frequency of FBD symptoms according to some studies. ADC, alternating diarrhea-constipation; FBD, functional bowel disorder

Study	Abdominal pain	Bloating	Diarrhea	Constipation	ADC
Our (*N* = 397)	68.8	67.3	2.8	61.7	5.3
Ono et al. 2018 (*N* = 547) [18]	37.84	36.01	18.65	32.54	
Diarra et al. 2011 (*N *= 104) [19]	97.1	51.9	22.1	45.2	9.7

In our study, diarrhea is particularly rare. This is explained by the non-inclusion of several subjects suffering from chronic diarrhea, whose incomplete exploration (absence of total colonoscopy with biopsies) meant that it was not possible to formally rule out another cause, notably microscopic colitis. Thus, an underestimation of the number of diarrhea cases in our study population cannot be excluded.

Among the associated digestive signs present in 314 patients (79.1%), dyspepsia was predominantly noted in 25.7% and GERD in 22.9%. These two pathologies are, in fact, frequently associated with FBD and, in particular, with IBS. Agréus et al. reported 87.5% functional dyspepsia in patients suffering from IBS [20]. In 2012, Pourhoseingholi et al. reported a frequency of 77.9% for dyspepsia and 74.7% for GERD, while Lovell and Ford found GERD in 42% of patients with IBS [21,22]. The simultaneous association of GERD, functional dyspepsia, and IBS is also reported in the literature [22,23].

Associated extra-digestive signs were present in 90 patients (22.67%). These included painful symptoms in 10.7% of cases, anxiety in 2.8%, and insomnia in 2.3%. These results echo those of Whitehead et al. in 2007, who reported 51 extra-digestive signs, each present in at least 1% of 3,153 patients with IBS [7]. Among these signs, 48 had a higher incidence in subjects with IBS compared to a non-IBS control population (OR > 1). Regarding anxiety-depressive disorders, our numbers are lower than those reported in the literature. Indeed, Cho et al. in 2011 reported anxiety in 38.6% of cases and depression in 38.6% of cases in patients with IBS [24]. Melchior et al. in 2020 found anxiety and depression in 43% and 14% of patients with IBS, respectively [25]. Our data probably underestimate this aspect due to the retrospective nature of the study, which did not allow us to ensure an exhaustive psychological evaluation in our patients. Overall, the psychosocial aspects of healthcare in our population do not receive sufficient attention.

Dietary guidelines were implemented in 94 patients (23.7%), including a high-fiber diet in 90 patients (22.7%), regardless of the type of FBD.

Dietary guidelines are part of the therapeutic options for FBD. The low-FODMAP diet is indicated and proven effective as first-line therapy in IBS, improving abdominal bloating, while a diet rich in soluble fiber, adequate fluid intake, and physical activity is useful in FBD with predominant constipation [26]. Several possible explanations exist for this trend, including the complexity of the low-FODMAP diet and limited resources in a developing-country context. Furthermore, the lack of research on FBD in our region raises questions about the adaptability of the dietary and lifestyle measures recommended in the literature to our specific context.

Psychological care was offered to 2 patients (0.5%).

The management of psychosocial comorbidities is an important part of therapy in FBD, which falls under disorders of gut-brain interaction [1,10,12]. However, such comorbidities were uncommon in our patients, with only 2.8% presenting with anxiety.

The median number of consultations was 3, with a mean of 2.67, a range of 1 to 27, and a standard deviation of 2.55. These results are comparable to those of Dapoigny et al., who reported a median of three consultations in a TFI population, and Lee et al., who reported a mean of 3.6 consultations with a general practitioner [27,28].

The lack of clinical improvement is often the cause of medical nomadism, a repetition of additional examinations and medication prescriptions, greatly increasing the cost of care.

There are some limitations to acknowledge about our study. Indeed, this is a retrospective study based on patient records. Selection bias cannot be ruled out. The records were inconsistently completed, and some data are therefore unavailable. Furthermore, this is a descriptive study with raw data, and potential relationships cannot be established without further correlational statistical analysis. Our results, therefore, constitute preliminary data, unprecedented in our context, on functional bowel disorders, and should serve as a basis for a prospective and analytical study on the subject.

## Conclusions

In our population, FBD mainly affects young adults, with a female predominance. Constipation-predominant IBS is the most common subtype of FBD. Treatment is symptomatic based on defoamer and antispasmodics, and a favorable outcome is more often noted in patients. Despite the size of the cohort, the retrospective and single-center nature of the study constitutes a limitation. Prospective and multicenter studies will, in the future, allow a better understanding of the specificities of functional bowel disorders in our context.
